# Xeno-free generation of new Yazd human embryonic stem cell lines (Yazd4-7) as a prior stage toward good manufacturing practice of clinical-grade raw materials from discarded embryos: A lab resources report

**DOI:** 10.18502/ijrm.v21i8.14017

**Published:** 2023-09-20

**Authors:** Fatemeh Hajizadeh-Tafti, Jalal Golzadeh, Fatemeh Akyash, Somayyeh-Sadat Tahajjodi, Ehsan Farashahi-Yazd, Hassan Heidarian-Meimandi, Behrouz Aflatoonian

**Affiliations:** ^1^Stem Cell Biology Research Center, Yazd Reproductive Sciences Institute, Shahid Sadoughi University of Medical Sciences, Yazd, Iran.; ^2^Department of Reproductive Biology, School of Medicine, Shahid Sadoughi University of Medical Sciences, Yazd, Iran.; ^3^Research and Clinical Center for Infertility, Yazd Reproductive Sciences Institute, Shahid Sadoughi University of Medical Sciences, Yazd, Iran.; ^4^Abortion Research Center, Yazd Reproductive Sciences Institute, Shahid Sadoughi University of Medical Sciences, Yazd, Iran.

**Keywords:** Derivation, Human embryonic stem cells, Human foreskin fibroblasts, Xeno-free, Good manufacturing practice, Mouse embryonic fibroblasts.

## Abstract

**Background:**

Xeno-free generation of human embryonic stem cells (hESCs) is important to prevent potential animal contaminations in culture for advanced cell-based therapeutic applications. Xeno-free production of hESCs is the first step for manufacturing clinical-grade hESC lines.

**Objective:**

To produce new hESC lines in xeno-free condition.

**Materials and Methods:**

This lab resources report was conducted at Stem Cell Biology Research Center, Yazd, Iran from 2019-2022. 4 new hESC lines from 11 (10 fresh and 1 frozen) donated surplus discarded human embryos were established. In this study, we report the xeno-free derivation of new Yazd hESC lines (Yazd4-7), without using immunosurgery, by culturing intact zona-free blastocysts obtained from discarded embryos onto the YhFF#8 cells as a feeder layer in a microdrop culture system. The pluripotency gene expression profile of the cell lines was assessed by reverse transcription polymerase chain reaction and the expression of specific surface markers was detected using immunofluorescent staining. In vitro differentiation was induced using embryoid body formation and gene expression profile of 3 germ layers and germ cells. Reverse transcriptase polymerase chain reaction was investigated to prove their pluripotent capacity.

**Results:**

In sum, we have been able to generate 4 new hESC lines (Yazd4-7) from 11 discarded embryos in xeno-free culture conditions using a micro drop culture system and YhFF#8 as a human source feeder layer.

**Conclusion:**

The outcome of this work can be the foundation for the future allogeneic cell-based therapeutic application using clinical grade good manufacturing practice-derived hESC derivatives.

## 1. Introduction

Human embryonic stem cells (hESCs) and human induced pluripotent stem cells are 2 major types of human pluripotent stem cells (hPSCs) that do not have any counterpart in vivo (1) and produce an in vitro cell culture artifact from different origins (embryos and somatic cells). These pluripotent stem cells have an unlimited self-renewal capacity in culture and pluripotency potential to differentiate the cells from all 3 germ layers and germ cells (2). These properties have made them good candidates as raw materials for producing allogeneic and autologous clinical-grade cells, tissues, and organs for cell-based therapeutic applications and regenerative medicine (3, 4).

Current progressions in understanding the clinical potential of hPSCs and their derivatives (cellular and extracellular) have raised concerns about the requirements for the derivation of clinical-grade hPSCs in reproducible, fully defined, xeno-free culture conditions (5).

In the case of hESCs, for generation of these pluripotent cells in a xeno-free condition should get rid of animal products such as animal-derived antibodies and mouse embryonic fibroblasts (MEFs), which are used in derivation during immunosurgery and expansion procedures. Successful derivation of hESCs in xeno-free conditions is the first step for producing these cells in a good manufacturing practice condition to generate cells and their derivatives in clinical-grade standards, which is critical in progressing stem cell therapies (6-8). To ensure the safety of the hESCs derivatives for the ultimate cell-based therapeutic cures, all potential risks, such as animal pathogens infection transmission by animal-derived materials, must be removed during the procedures (9, 10). Therefore, all phases of the manufacturing of hESC lines should prevent the usage of animal cells and animal-origin materials while doing derivation, expansion, and differentiation of hESC lines in culture medium and processes. Otherwise, such xeno-contaminated hESCs and their derivatives are typically known as inappropriate raw materials and final products for transplantation due to the hazards of zoonosis infections which may not be rejected by the immune system (11, 12).

One of the sources of animal pathogen infections is MEFs, which have been used as feeder layers during the derivation and expansion of hESCs. Alternatively, several human-originated feeder layers exist such as fetal muscle, fetal skin, adult fallopian tubal epithelial cells, and foreskin fibroblasts (13, 14). The hESC lines derived using various sources of human feeder layers display similar features to those generated on MEFs, with distinct morphological characteristics being small and roundish with a high ratio of nucleus to cytoplasm while expressing specific markers (15).

Moreover, it has been demonstrated that hESC lines cultured on human feeders are more feasible to expand with less spontaneous differentiation than those cultured in feeder-free culture conditions (16, 17). The other way of animal pathogen transmission during the generation of hESC lines are antibodies developed in animals against human antigens used during immunosurgery procedures for the derivation of hESCs. It has been reported that by using a microdrop culture system, hESC lines can be derived without the need of immunosurgery (14, 18). In the current study, we are reporting the generation of new Yazd hESC lines (Yazd4-7) from discarded embryos (19) using a microdrop culture system (14, 18) onto the Yazd human foreskin fibroblasts #8 (20) feeder layer. The cells were characterized using specific markers following derivation and expansion. Their pluripotency capacity was evaluated by applying in vitro differentiation in monolayer culture and embryoid body (EB) formation as the most obvious alternative to the teratoma assay (21).

This study aimed to set up the generation of new hESC lines in xeno-free conditions before establishing a good manufacturing practice construction to generate clinical-grade hESC lines and their derivatives for future regenerative medicine applications.

## 2. Materials and Methods

This lab resources report was conducted at Stem Cell Biology Research Center, Yazd, Iran from 2019-2022. 4 new hESC lines from a total of 11 (10 fresh and 1 frozen) donated surplus discarded human embryos were established.

Chemicals and culture media (with their supplements) were obtained from Sigma Aldrich (Poole, UK) and Invitrogen-Gibco (UK) unless otherwise stated.

### Preparation of human feeders (YhFF#8) in microdrops

For the derivation of new Yazd hESC lines (Yazd4-7) in a xeno-free condition, Yazd human foreskin fibroblasts batch 8 (20) were used as human feeder following mitotically inactivation with 4 hr of mitomycin-C treatment. Microdrops of feeders were prepared as explained elsewhere (14, 18).

### Generation of new Yazd hESC lines (Yazd4-7)

To prevent using animal antibodies for inner cell mass isolation in immunosurgery procedure, whole zona-free blastocyst culture into the microdrop culture system was applied. The zona-pellucida (ZP) was removed by treating the human embryos with pronase (2 mg/ml; Calbiochem, USA) for 3-5 min, depending on the ZP thickness and stiffness. Followed by washing, the ZP-free human embryos were cultured into the microdrops coated with mitotically inactivated YhFF#8 cells as a human feeder layer. Depending on different factors, mainly, the blastocyst quality, between days 3-8 following seeding ZP-free blastocysts in microdrops of YhFF#8, the initial outgrowths were observed. Within some of the initial outgrowths, distinguished clumps of hESCs (18) were observed, then by using pulled glass pipettes they were mechanically cut and pasted into the new microdrops of feeders. Following 2 or 3 passages, the colonies appeared flattened, and hESCs were observed in a unique morphological feature. The new lines were expanded using mechanical passaging in microdrop, and their EB formation capacity was evaluated using nonadherent culture conditions similar to what was explained elsewhere (14). New cell lines were frozen using the vitrification method for producing master and working cell banks for each cell line.

### Characterization of new Yazd hESC lines (Yazd4-7)

The expression of pluripotency markers and genes by the newly derived Yazd hESC lines (Yazd4-7) was assessed using immunofluorescent (IF) staining and reverse transcriptase polymerase chain reaction (RT-PCR). IF and RT-PCR were performed according to the manufacturer's instructions (14). Antibodies used for IF are listed in table I. RT-PCR was done using the cDNA synthesis and primers for different genes (Table II). The chromosomal content of the cells was evaluated using G-banding karyotype as explained elsewhere (14). The pluripotency capacity of the cell lines was evaluated by differentiation in vitro assay (21) using spontaneous differentiation in monolayer culture and EB formation similar to that explained elsewhere (14). Primers that were used to evaluate pluripotency are listed in table II.

**Table 1:** List of primary and secondary antibodies


**Primary antibody**			**Secondary antibody**		
**Name**	**Dilution**	**Catalog number**	**Type**	**Dilution**	**Catalog number**
**TRA-1-60**	Abcam ab16288	Rabbit anti-mouse IgG+IgM+IgA H&L (FITC)	Abcam ab8517
**MC813-70 (SSEA-4)**	Santa Cruz sc-21704		
**TRA-2-49**	1:100	Abcam ab17973	Rabbit anti-mouse IgG H&L (TR)	1:200	Abcam ab6726

**Table 2:** List of primers used for RT-PCR


**Status**	**Gene**	**Forward primer (5 ${}^{'}$ -3 ${}^{'}$ )**	**Reverse primer (5 ${}^{'}$ -3 ${}^{'}$ )**	**Prod size (bp)**
	*NANOG *(NM_024865.3)	CCCCAGCCTTTACTCTTCCTA	CCAGGTTGAATTGTTCCAGGTC	97
	*POU5F1 *(NM_002701.5)	GGTTGAGTAGTCCCTTCGCA	TAGCCAGGTCCGAGGATCA	174
**Pluripotency**	*SOX2 *(NM_003106.3)	AGGACTGAGAGAAAGAAGAGG	GAGAGAGGCAAACTGGAATC	163
		*PAX6* * * (NM_000280.4) * *	AGATTCCTATGCTGATTGGTGAT	AGGAGGAAGTGTTTTGCTGGA	139
	**Ectoderm**	*TUBB3* (NM_006086.3)	ACCAGATCGGGGCCAAGT	GGCACGTACTTGTGAGAAGAGG	142
		*DES *(NM_001927.3)	GTGCATGAAGAGGAGATCCGT	ATGTTCTTAGCCGCGATGGT	143
**Mesoderm**	*TBXT *(NM_003181.3)	GGCGCGAGAACAGCACTA	CCAAGACTGTCCCCGCTC	117
	**Endoderm** * *	*SLC2A1* (NM_006516.2)	TTGGCTCCGGTATCGTCA	CTCAGATAGGACATCCAGGGTA	172
	*VASA *(NM_024415.2) * *	ACAGATGCTCAACAGGATGTTCC	CCCTTTCTGGTATCAACTGATGCA	119
**Germ cell** * *	*DAZL *(NM_001351.3)	CCTTGTCACCCGCTCTTG	ATTTGCAGTAGACATGATGGCG	183
	**Housekeeping** * *	*B2M *(NM_004048.2)	AGATGAGTATGCCTGCCGTG	TGCGGCATCTTCAAACCTC	106

### Ethical considerations

This study was ethically approved by the Ethics Committee of the Shahid Sadoughi University of Medical Sciences, Yazd, Iran (Codes: IR.SSU.REC.1394.104 and IR.SSU.REC.1394.105). All embryos were coded to ensure anonymity. Moreover, the derivation and characterization procedures of the cell lines were done according to the guidelines and the ethical considerations. Informed written consent was obtained from every infertile couple.

## 3. Results

### Generation of xeno-free hESCs (Yazd4-7)

**Figure 1:**
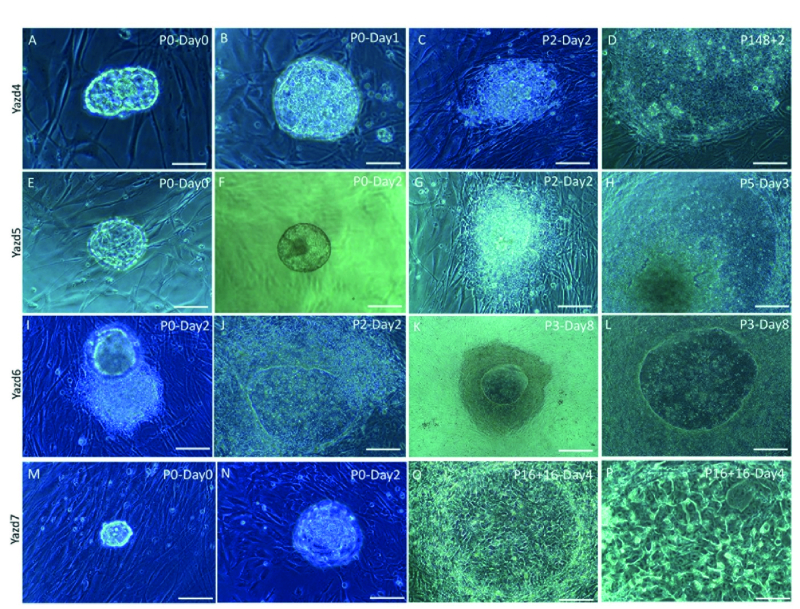
Generation of new xeno-free Yazd hESC lines (Yazd4-7) from zona-free blastocysts cultured to the YhFF#8 feeder in a microdrop culture system. From the initial outgrowth to the earliest distinct colony of each cell, lines (Yazd4: A-D, Yazd5: E-H, Yazd6: I-L, Yazd7: M-P) were monitored and recorded by passage number.

From 11 discarded human embryos, 4 new hESC lines (Yazd4-7) were derived on neonatal human foreskin fibroblasts (20) as feeders using microdrop culture conditions (14, 18). The establishment of each cell line is summarized in figure 1. Master and working cell banks were established for each cell line using vitrification (14). Cell lines were thawed to check their survival and expansion capacity following vitrification.

### Characterization of the xeno-free derived Yazd hESC lines (Yazd4-7)

4 new hESC lines were derived in a xeno-free condition using YhFF#8 as a human feeder and microdrop culture instead of immunosurgery. Each cell line was characterized using specific markers and assessment of their gene expression profile during undifferentiated and differentiated conditions to prove their pluripotency. The cells in an undifferentiated state showed distinct morphological characteristics with a high nucleus: cytoplasm ratio with distinct nucleoli (16).

**Figure 2:**
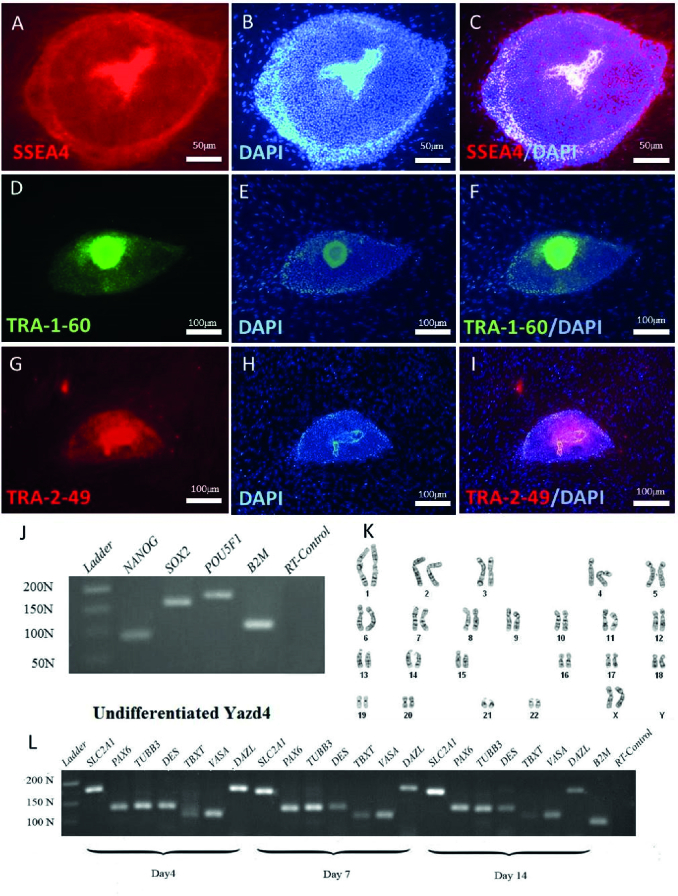
Characterization of Yazd4 hESC line expression of pluripotency markers SSEA4 (A-C), TRA-1-60 (D-F), and TRA-2-49 (G-I) was assessed by IF staining. *NANOG*, *SOX2,* and *POU5F1 *expression aspluripotency genes (J) was evaluated using RT-PCR undifferentiated Yazd4 hESCs. Normal 46, XX karyotype for Yazd4 hESC line (K) was detected by G-banding. Differentiation of Yazd4 hESC line into 3 embryonic germ layers (ectoderm: *PAX6, TUBB3*, mesoderm: *DES, TBXT*, endoderm: *SLC2A1*) and germ cells (*VASA* and *DAZL*) using EB formation after 4, 7 and 14 days (L).

IF results have indicated that undifferentiated Yazd4 hESCs express specific surface markers such as SSEA4 (Figure 2A-C), TRA-1-60 (Figure 2D-F), and TRA-2-49 (Figure 2G-I), while RT-PCR data confirmed the expression of pluripotency genes *NANOG*, *SOX2*, and *OCT4/POU5F1* (Figure 2J) by the cells. Moreover, G-banding karyotyping showed that Yazd4 cells contained normal 46, XY chromosomal content (Figure 2K). As it is shown in figure 2L, differentiation in vitroassay (15) by EB formation and gene expression analysis for 3 germ layers (ectoderm*:* *PAX6*, *TUBB3*; mesoderm: *DES*, *TBXT*; endoderm: *SLC2A1* and germ cells *DDX4/VASA *and *DAZL*).

**Figure 3:**
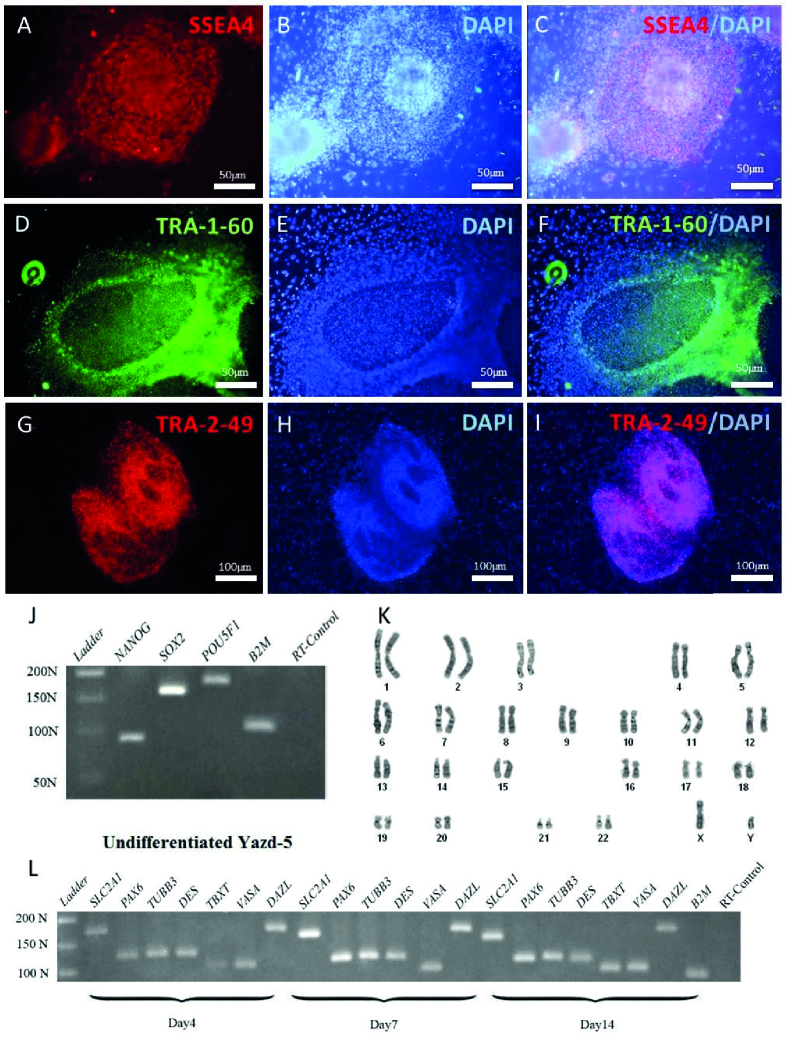
Characterization of Yazd5 hESC line. Positive expression of pluripotency markers SSEA4 (A-C), TRA-1-60 (D-F), and TRA-2-49 (G-I) was tested using IF. RT-PCR data showed the expression of *NANOG*, *SOX2,* and *POU5F1 *aspluripotency genes (J) in undifferentiated cells. Normal 46, XY karyotype for Yazd5 hESC line (K) was shown using G-banding. Differentiation by EB formation and gene expression analyzing for 3 germ layers (ectoderm: *PAX6, TUBB3*, mesoderm: *DES, TBXT*, endoderm: *SLC2A1*) and germ cells (*VASA* and *DAZL*) (L).

Undifferentiated Yazd5 colonies showed positive expression for surface markers such as SSEA4 (Figure 3A-C), TRA-1-60 (Figure 3D-F), TRA-2-49 (Figure 3G-I), and pluripotency genes such as *POU5F1*,* NANOG*,and* SOX2* (Figure 3J). The karyotype of Yazd5 hESCs was normal 46, XX as revealed by a G-banding (Figure 3K). The pluripotency capacity of Yazd5 cells was proven by in vitro differentiation assay to 3 embryonic germ layers (ectoderm: *PAX6*, *TUBB3*; mesoderm: *DES*, *TBXT*; endoderm: *SLC2A1*) and germ cells (*VASA* and *DAZL*) using EB formation after 4, 7, and 14 days (Figure 3L).

**Figure 4:**
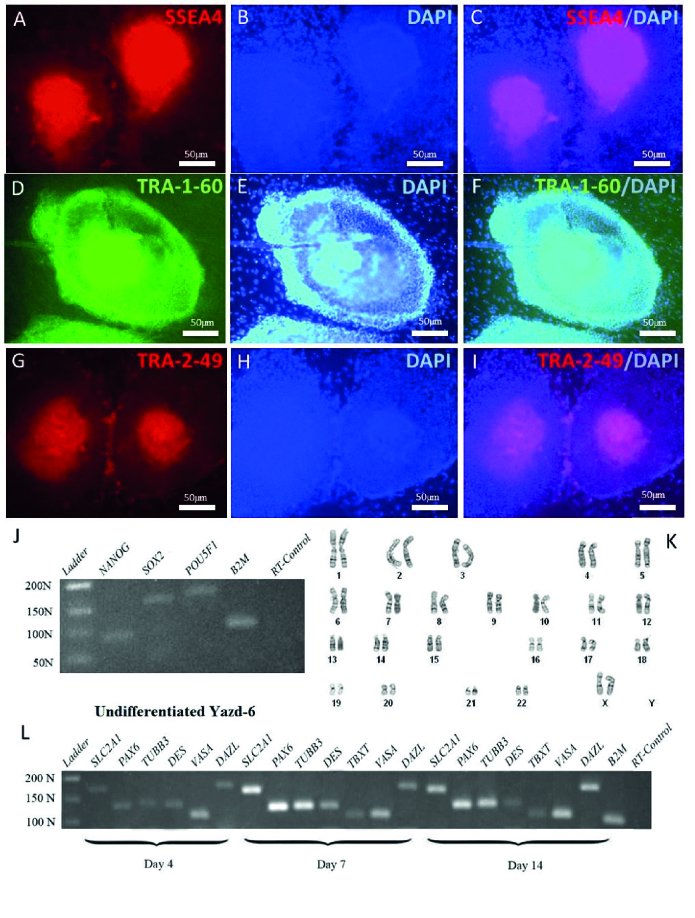
Characterization of Yazd6 hESC line. The New Yazd6 hESC line was characterized with specific markers such as SSEA-4 (A-C), TRA-1-60 (D-F), and TRA-2-49 (G-I), and pluripotency genes including *NANOG, SOX2,* and *POU5F1* (J). Yazd6 hESC line was a normal 46, XX cell line (K). Furthermore, the differentiation potential of the cell line with EB formation culture was determined using the RT-PCR technique for 3 embryonic germ layers and germ cells specific genes (L).

Similarly, undifferentiated Yazd6 hESC colonies displayed specific characteristics such as SSEA4 (Figure 4A-C), TRA-1-60 (Figure 4D-F), and TRA-2-49 (Figure 4G-I) while expressing pluripotency genes *POU5F1*, *NANOG*, and *SOX2* (Figure 4J). Yazd6 hESC line was a normal 46, XX cell line as shown by G-banding (Figure 4K). Following EB formation after 4, 7, and 14 days Yazd6 cells showed positive expression of 3 embryonic germ layers (ectoderm: *PAX6*,* TUBB3*; mesoderm: *DES*, *TBXT*; endoderm: *SLC2A1*) and germ cells (*VASA* and *DAZL*) and shown in Figure 4L.

**Figure 5:**
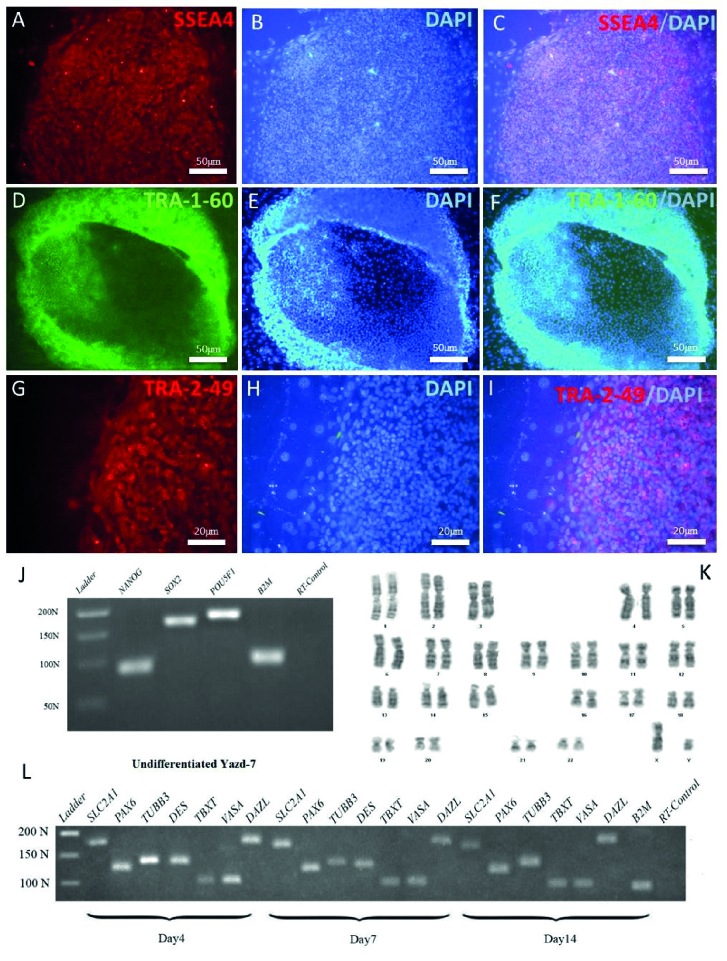
Characterization of Yazd7 hESC line expression of pluripotency markers SSEA4 (A-C), TRA-1-60 (D-F), and TRA-2-49 (G-I) was tested using IF. RT-PCR data showed positive *NANOG, SOX2,* and *POU5F1* expression as pluripotency genes (J) in undifferentiated cells. Normal 46, XY karyotype for Yazd7 hESC line (K) was shown using G-banding. RT-PCR was used to detect the 3 embryonic germ layers and specific genes of germ cells with EB formation (L).

Likewise, Yazd7 hESCs showed positive expression of SSEA4 (Figure 5A-C), TRA-1-60 (Figure 5D-F), and TRA-2-49 (Figure 5G-I) markers and *POU5F1*, *NANOG*, and *SOX2* as pluripotency genes (Figure 5J) while displaying normal 46, XY karyotype (Figure 5K). Cells within differentiated EBs of Yazd7 have shown the expression of 3 germ layers (ectoderm: *PAX6*, *TUBB3*; mesoderm: *DES*, *TBXT*; endoderm: *SLC2A1*) and germ cells (*VASA *and *DAZL*) as confirmed by RT-PCR (Figure 5L).

## 4. Discussion

4 new hESC lines (Yazd4-7) were generated using discarded embryos (19) in a xeno-free condition using YhFF#8 as a human feeder and established following characterization with specific markers and genes in undifferentiated status. The pluripotency of the new hESC lines was assessed by in vitrodifferentiation and gene expression profile analysis for 3 germ layers and germ cells (21). Master and working cell banks were established using vitrification method (14).

Since the first derivation of hESCs by Thomson and colleagues (22, 23), scientific societies, and policymakers in health (24) were concerned regarding the safety of hESCs and their derivatives besides their effectiveness during their manufacturing procedures (25). Conventionally hESCs were derived using immunosurgery onto the mitotically inactivated MEFs as feeder layer (8, 12, and 17) due to the presence of the animal antibodies and mouse feeders, there was the risk of animal source pathogens infection transmission into the products (26).

The alternative process for immunosurgery is using whole zona-free blastocyst seeding onto the feeders in a microdrop culture system (14, 18), which was used to derive xeno-free Yazd hESCs (Yazd4-7).

On the other hand, different sources of human cells are used as feeder layers to replace MEFs. Human foreskin fibroblasts (hFFs) isolated from infant foreskin are the most popular source of human feeders, which have been used for expansion (14) or derivation of hESCs (27, 28). Besides the xeno-free concern, hFFs lifespan is longer than MEFs before entering the decline phase in growth and propagation and starting senescence (28, 29).

Some groups produce hFFs in their labs (20), and others buy the commercially available hFFs (27) as human source feeder cells to propagate and derive hESCs. Here, we have used Yazd human foreskin fibroblasts #8 (YhFF#8), produced in our center (20), as a human feeder source. Before using YhFF#8 as a feeder for the derivation procedure, their capacity to support the expansion of the hESCs in an undifferentiated state was tested using already derived hESCs on MEFs (Yazd1-3; 8).

In sum, 4 new hESCs (Yazd4-7) were derived in xeno-free conditions avoiding animal products during the manufacturing procedure to set up the first step of the generation of clinical-grade hESCs for future applications in regenerative medicine. The cell lines were characterized morphologically as they have shown a high nucleus: cytoplasm ratio and dominant nucleoli (16).

Furthermore, cells displayed specific markers and pluripotency gene expression profiles, as shown in the results. All the cell lines contained normal chromosomal content. In vitro differentiation assay (21) using EB formation and gene expression assessment for 3 germ layers and germ cells indicated the pluripotency capacity of the xeno-free Yazd hESC lines (Yazd4-7).

Moreover, the rate of the hESC lines derivation success from discarded embryos (4 cell lines from 11 embryos; ∼ 37%) was efficiently and sufficiently higher than our previous report, 18.75% (8), which might be due to expertise and quality of the feeder. Master and working cell banks were established using vitrification similar to what was done for our previously produced cell lines (Yazd1-3; 8).

## 5. Conclusion

Following the establishment of Yazd human foreskin fibroblast lines (YhFF#8, #17, and #18; 14) and testing them as a feeder layer for propagation and expansion of Yazd hESCs (Yazd1-3; 8), new hESC lines (Yazd4-7) were generated on YhFF#8 feeder layer in a microdrop culture condition with KOSR/HES without immunosurgery as a xeno-free condition. Xeno-free derivation of hESCs, as the first step of clinical-grade manufacturing of hESCs, will lend itself toward producing good manufacturing practice -grade hESCs in its suitable hygiene construction.

##  Conflict of Interest

The authors declare that there is no conflict of interest.
